# Informing Stewardship Measures in Canadian Food Animal Species through Integrated Reporting of Antimicrobial Use and Antimicrobial Resistance Surveillance Data—Part I, Methodology Development

**DOI:** 10.3390/pathogens10111492

**Published:** 2021-11-16

**Authors:** Agnes Agunos, Sheryl P. Gow, Anne E. Deckert, Grace Kuiper, David F. Léger

**Affiliations:** 1 Center for Foodborne, Environmental and Zoonotic Infectious Diseases, Public Health Agency of Canada, Guelph, ON N1H 7M7, Canada; anne.deckert@phac-aspc.gc.ca (A.E.D.); david.leger@phac-aspc.gc.ca (D.F.L.); 2 Center for Foodborne, Environmental and Zoonotic Infectious Diseases, Public Health Agency of Canada, Saskatoon, SK S7N 5B4, Canada; sheryl.gow@phac-aspc.gc.ca; 3 Department of Environmental and Radiological Health Sciences, Colorado State University, Fort Collins, CO 80523, USA; grace.kuiper@colostate.edu or

**Keywords:** integration, summarized reporting, antimicrobial use, antimicrobial resistance, stewardship, metric, indicator

## Abstract

This study explores methodologies for the data integration of antimicrobial use (AMU) and antimicrobial resistance (AMR) results within and across three food animal species, surveyed at the farm-level by the Canadian Integrated Program for Antimicrobial Resistance Surveillance (CIPARS). The approach builds upon existing CIPARS methodology and principles from other AMU and AMR surveillance systems. Species level data integration involved: (1) standard CIPARS descriptive and temporal analysis of AMU/AMR, (2) synthesis of results, (3) selection of AMU and AMR outcomes for integration, (4) selection of candidate AMU indicators to enable comparisons of AMU levels between species and simultaneous assessment of AMU and AMR trends, (5) exploration of analytic options for studying associations between AMU and AMR, and (6) interpretation and visualization. The multi-species integration was also completed using the above approach. In addition, summarized reporting of internationally-recognized indicators of AMR (i.e., AMR adjusted for animal biomass) and AMU (mg/population correction unit, mg/kg animal biomass) is explored. It is envisaged that this approach for species and multi-species AMU–AMR data integration will be applied to the annual CIPARS farm-level data and progressively developed over time to inform AMU–AMR integrated surveillance best practices for further enhancement of AMU stewardship actions.

## 1. Introduction

Disease mitigation strategies, such as infection prevention and control (IPC), good management practices (GMP) and enhanced disease detection, are utilized in Canadian food animal species, including broiler chickens, grower-finisher (GF) pigs and turkeys. Pertinent species-specific diseases are monitored through provincial and regional networks of private practitioners, animal disease diagnostic laboratories and government partners [1,2,3,4]. For the prevention or treatment of these diseases, there are approved antimicrobials [5,6], however, the global call for antimicrobial use (AMU) reduction to contain antimicrobial resistance (AMR) [7] has limited antimicrobial options for the prevention of bacterial diseases in the Canadian poultry industry. Managing the clinical and economic implications of these pathogens in animal production settings through IPC [7] or GMP, including biosecurity, in addition to enhanced monitoring and surveillance of AMU and AMR, are recommended to reduce the need for antimicrobials and the containment of AMR [7,8,9].

Antimicrobial stewardship pertains to “the multifaceted approaches required to sustain the efficacy of antibiotics and minimize the emergence of resistance” [10]. Surveillance of AMU is indispensable for tracking the impact of AMU stewardship measures in animal production. Analyzing, reporting and communication of surveillance data has advanced in the last decade. This is, in part, due to the development of AMU metrics (technical units of measurements such as frequency of use, mg of antimicrobial active ingredients and number of defined daily doses in animals (DDDvet)) and AMU indicators (a metric in relation to a denominator, such as population and weight or days at risk) [11,12,13]. Internationally, there are recognized weight-based indicators for describing AMU. These include the European Surveillance for Veterinary Antimicrobial Consumption’s (ESVAC) mg of antimicrobial active ingredient per population correction unit (mg/PCU), [14] used for the harmonized reporting of AMU in the European Union/European Economic Area (EU/EEA), and the OIE’s mg/kg animal biomass [15], used for reporting the global AMU data. Dose-based indicators such as Treatment Incidence [TI_100_ and TI_1000_], DDDvet per PCU and other derivatives of these indicators (e.g., annualized estimates such as DDDvet/Year) are also utilized by regional and national AMU monitoring systems and in AMU research [13,16,17,18,19,20,21,22,23]. Unfortunately, there is no single AMU metric or indicator that can address all the objectives of an AMU surveillance program or study. For example, surveillance objectives may include the need to monitor trends over time, to compare AMU between animal species and between geographical locations, to evaluate AMU reduction or stewardship measures, to understand the impact of AMU in relation to AMR or to compare AMU and AMR with international surveillance systems. As such, AMU monitoring systems have developed AMU indicators to appropriately fit their circumstance, including the type of data available (qualitative vs. quantitative), the stage in the AMU distribution chain where data are collected (farm, veterinary practice, feed mills), the mechanism for data collection (voluntary vs. mandatory reporting), and the AMU objectives of the program. The Canadian Integrated Program for Antimicrobial Resistance Surveillance (CIPARS) sentinel farm surveillance [24], and several researchers, have compared how AMU indicators relate to each other and how input parameters could change the interpretation [17,25,26,27]. In order to describe the various aspects of AMU and address various AMU surveillance objectives, it is therefore necessary to include multiple complimentary indicators.

Across Canada, CIPARS routinely monitors trends in AMR in select bacteria isolated from humans, animals and animal products along the food chain and AMU in human, animal and food sources across Canada. CIPARS Farm Surveillance focuses on surveillance of food animal species through a network of sentinel veterinarians and producers [28]. The CIPARS farm component was designed to collect annual data from a network of sentinel veterinarians and producers (i.e., longitudinal data collection) and started in GF pigs in 2006, and progressively expanded to broiler chicken (2013), turkey (2016) and feedlot beef (2019, not included in this paper). The animal species surveyed are essential to Canadian food security and economy. In terms of per capita consumption, chicken ranks first, pork second, beef third and turkey fourth [29].

CIPARS reports trends in AMR in select bacterial species (% resistance, number of classes in resistance patterns), trends in AMU in mg/PCU and number of defined daily doses in animals using Canadian standards (nDDDvetCA) per 1000 animal-days at risk (TI_1000_) [28]. Recently, CIPARS explored how AMU indicators (routine and exploratory indicators) relate to each other, and discovered a high correlation between the different weight-based (mg/PCU vs. mg/kg animal biomass) indicators and the different dose-based indicators (nDDDvetCA/1000 animal-days at risk vs. nDDDvetCA/PCU), respectively [24]. CIPARS also previously explored the utility of AMR indicators for AMU–AMR data integration using routine AMR outcomes (% resistance) and % resistance adjusted for PCU [30]. Percentage of resistance adjusted for PCU accounts for species-specific AMR prevalences and the fluctuations in animal biomass over time and is used for summarized reporting of AMR findings in the EU/EEA [31,32]. This indicator is not currently used by CIPARS, since the biomass from sentinel farms has been relatively stable.

CIPARS species-specific questionnaires collect AMU, select biosecurity, diseases diagnosed and preventive health information. Despite obtaining a variety of data, trends in clinical impressions of the veterinarians completing the questionnaires on common diseases and relevant preventive health measures in relation to AMU have not yet been fully explored. It is envisaged that incorporating health information into AMU–AMR surveillance analysis could contribute to the enhancement of commodity-specific AMU stewardship programs. In the context previously described, the objective of this study is to further develop various aspects of AMU and AMR integration methodologies within each species. This objective incorporates several steps including (a) assessment of AMU and AMR surveillance findings through routine analysis, synthesis and the identification of AMU and AMR outcomes based on public and animal health importance, (b) the selection of the most appropriate AMU indicator for comparison of AMU between species (trends and levels of AMU) and for assessing potential AMU and AMR associations, (c) the investigation of analytic options for evaluating the relationship between AMU and AMR and (d) the exploration of the utility of animal health data to understand potential implications of changes in AMU trends. Through taking this structured approach for data integration, the study will inform surveillance analysis and best practices on how to better synthesize and report diverse information collected from farm programs. For the purposes of this paper, integration pertains to the reporting of at least two surveillance data types (e.g., AMU–AMR, AMU-animal health) and summarized reporting pertains to the combined data from across the three animal species. Here we present the methodology (existing and exploratory) by thematic areas (AMU, AMR, AMU–AMR analysis, animal health context) and discuss the rationale for the preferred approaches. In Section 2, the methodology development for species-level AMU–AMR data integration is described and the utility of syndromic data discussed. In Section 3, methodology development for multispecies-level AMU–AMR, summarized reporting and analytical methods derived from Section 2 are presented.

## 2. Methodology Development for Species-Level AMU–AMR Data Integration

**Figure 1:**
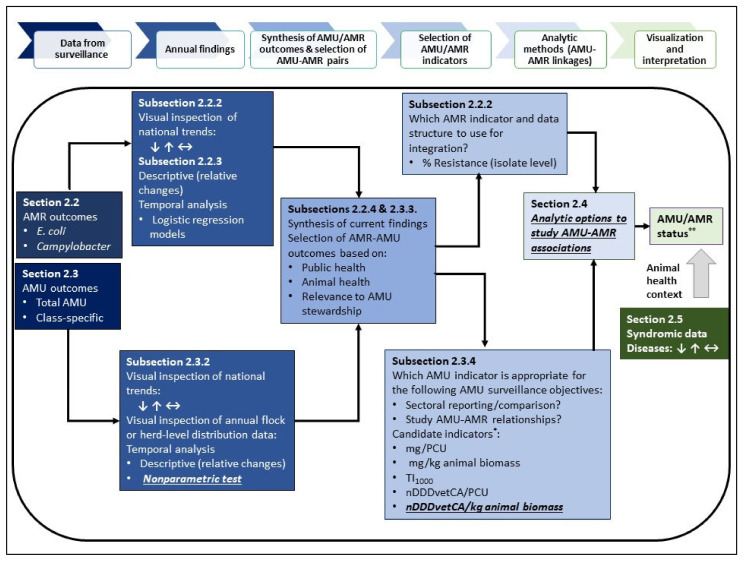
Approach to species-level integration of farm-level antimicrobial use and resistance data and utility of the syndromic health data for context. AMU-antimicrobial use; AMR-antimicrobial resistance. Arrows indicate the directionality of the shifts in the national, five year AMU, AMR and animal health data used in this study. Bold and underlined items represent exploratory analysis. * Candidate indicators included those that are routinely used by CIPARS and exploratory AMU indicators. ** Species-level status of AMU and AMR.

The methodological steps (routine and exploratory) for AMU and AMR data integration are outlined in Figure 1. From this point forward, each subsection provides a brief description of the method used, the results from either exploratory analysis or expansion of current CIPARS methodology (i.e., the basis for the selection of a new approach or analysis) and the rationale as to why a particular method or analysis was selected for further data integration. The selection of AMU/AMR outcomes for species-level integration and visualization, AMU–AMR association analysis and the utility of animal health outcomes for contextualizing the AMU–AMR findings are also highlighted. Broiler chicken and GF pig data collected between 2015 and 2019 and turkey data collected between 2016 and 2019 were the data sources for this study. All input parameters included in the various analysis/exercise are summarized in Appendix A by year of study. For the purposes of this paper, synthesis pertains to the method of synthesizing results from the CIPARS Farm AMU–AMR surveillance program and interpreting these results within the context of national and global recommendations to contain AMR.

### 2.1. General Surveillance Methods

#### 2.1.1. Study Design, Data and Sample Collection

Overview: The CIPARS farm component is a sentinel-based surveillance program that collects samples and data from a network of veterinary practices and their producers [28]. In GF pigs, the same sampling units (farm, barns or pens) are visited every year. However, in broiler chicken and turkey farms, the same sampling units may not be available for sampling every year because of logistical reasons (i.e., rapid turnover of flocks and availability of the same barn/pen at the time of sampling). In this case, the veterinary practices collect samples/data from other farms within their practice/producer network. An average of 17 poultry veterinarians and 20 swine veterinarians participate in the CIPARS farm AMU/AMR surveillance program each year. Flocks and herds are located in major poultry (five provinces) and swine (four provinces) producing provinces in Canada. A species-specific questionnaire, developed in consultation with sectoral representatives, is used to collect AMU, farm-level demographics, biosecurity and the animal health status of the flock or herd. Data to complete the survey may be obtained from a variety of sources, such as on-farm food safety records or observations at the time of farm visit. The veterinarian or veterinary field staff administers the questionnaire to the producer or farm staff during the sampling visit. 

The number of flocks or herds required for each commodity being studied (i.e., broiler chickens, turkeys and GF pigs) within the surveillance system was determined using the methods described elsewhere [20] and the WinEpi program [33].

- Broiler chickens: during the first surveillance year (2013), 100 flocks were allocated nationally and distributed across the five major broiler chicken producing provinces where the number of flocks allocated per province was based on the provinces’ relative contribution to national chicken production. However, in subsequent years, the number of flocks per province was adjusted to satisfy a requirement of collaborative surveillance system, FoodNet Canada, to have a minimum of 30 flocks within a FoodNet Canada Sentinel site [34]. This adjustment resulted in >135 flocks being sampled per year. Quantitative AMU methodology was developed within the first three years of surveillance (i.e., mg/PCU); these AMU estimates enabled validation of national flock allocations. From the 2705 (national three-year (2013–2015) mean) broiler chicken producers in Canada [35], approximately 133 broiler chicken flocks were required per year based on the three-year (2013–2015) mean mg/PCU of 147 [36], an accepted error of 5% and a 90% confidence interval (CI).
- Turkeys: 56 flocks from a national total of 548 turkey producers (2010 data) [37] were required per year based on the first three-years (2013–2015 pilot project) mean mg/PCU of 67 [30], accepted error of 5% and a 95% CI. These flocks were allocated in the four major turkey producing provinces. Similar to broiler chickens, additional flocks were added in FoodNet Canada sentinel sites which increased the total number of flocks to greater than 90 per year.
- GF pigs: herds were distributed across the five major pork producing provinces in proportion to each province’s contribution to the number of GF operations nationally. Participating veterinarians were directed to enroll herds that were representative of the demographics of their swine practice with respect to size and management type. Unless they withdrew from the program, enrolled herds participated in subsequent years. Between 2006 and 2019, the number of herds participating nationally ranged from 85 to 108. In 2017, questionnaire and database refinements yielded more complete quantitative AMU data (i.e., inclusion of water and parenteral AMU in addition to feed), thereby allowing total AMU to be calculated and validation of enrolled herd numbers to be performed. Based on the three-years mean of 101 mg/PCU and mean of 7973 swine herds nationally [38] with an accepted error of 5% and 95% CI, 96 herds were required per year.

In poultry species (from this point forward, poultry pertains to both broiler chickens and turkeys), four pooled samples were collected from the randomly selected sampling unit (i.e., barn, floor or pen) within a sentinel farm. One pool comprised of at least 10–15 droppings from individual birds (40 to 60 total/flock). As described elsewhere [39], the total number of birds per sampling unit was based on the formula for detection of AMR (e.g., tetracycline) in a population of 1000 or more individuals (*n* = ln α/88 ln (1-minimum expected AMR prevalence), α = 0.05) [40]. This barn-level sampling strategy was based on two studies in Ontario, Canada: (1) baseline study of *Salmonella* and *Campylobacter* prevalence [41] and (2) AMU–AMR pilot study comparing antibiotic-free, organic and conventional broiler chicken flocks [42].

In GF pigs, pooled fecal samples were collected from five quadrants in each of six pens of close-to-market pigs (>80 kg) on each sentinel herd in order to both maximize the number of individual pigs represented in the composite samples as described by Dunlop [43] and to assess resistance in animals as close as possible to the total days of risk for antimicrobial exposure.

#### 2.1.2. Statistical Analysis 

All statistical analyses were performed in SAS Version 9.4, Stata/SE Version 16.1 or Microsoft Excel (Microsoft Office Professional). All input parameters used in this paper are summarized in Appendix A.

### 2.2. AMR Data Component

Overview: the CIPARS farm component follows a standardized approach for sample collection on farm as previously described. All samples are shipped in refrigeration temperature and tested at the National Microbiology Laboratory (NML) in St. Hyacinthe, Québec. Farm samples are processed according to routine CIPARS microbiological methods on bacterial recovery, further characterization of bacterial isolates (e.g., serotyping of *Salmonella*, speciation of *Campylobacter*) and antimicrobial susceptibility testing [28]. Antimicrobial susceptibility testing is methodologically similar to that of the United States’ National Antimicrobial Resistance Monitoring System (NARMS) [44,45]. 

#### 2.2.1. Bacterial Isolation and Susceptibility Testing 

The recovery of *E. coli* and *Campylobacter* from fecal samples was performed according to routine CIPARS/FoodNet Canada methodology described elsewhere [28]. For AMR testing, the minimum inhibitory concentration (MIC) values for *E. coli* and *Campylobacter* were determined by automated broth microdilution using the Sensititre system (Sensititre^TM^ Trek Diagnostic Systems Ltd., West Sussex, UK) and the United States National Antimicrobial Resistance Monitoring System (NARMS) Gram-negative (CMV4AGNF containing fourteen antimicrobials belonging to eight classes) and CAMPY plates (nine antimicrobials belonging to seven classes), respectively [28]. 

#### 2.2.2. AMR Indicator Analysis

For this paper, the term indicator pertains to the AMR measurements used including: (1) % resistance (number of resistant isolates divided by total bacteria recovered), and (2) % resistance adjusted for species-specific total resistant isolates, total bacteria recovered and animal biomass (described in detail in Section 3). For studying AMR outcomes (outcome examples are resistance to single or homologous antimicrobials such as gentamicin resistance (GEN-R), susceptibility to all antimicrobials tested, or resistance to ≥three antimicrobial classes included in the panel), the indicator organism, *E. coli* was selected, which is consistent with many other surveillance systems. *E. coli* was chosen for this purpose because of the robustness of the data and its reliability for ongoing AMR monitoring. In the CIPARS dataset, recovery of *E. coli* was consistently >99%. *Campylobacter* was the second bacterial species used because of the importance of a specific AMR outcome, ciprofloxacin resistance (CIP-R) and azithromycin resistance (AZM-R). For the sake of completeness, it should be noted that CIPARS also routinely tests *Salmonella* spp. for antimicrobial susceptibility, but because of the impact of serovar variations on AMR, this organism was not included in this study. 

Bacterial isolation and AMR data for *E. coli* and *Campylobacter* were extracted from the Public Health Agency of Canada’s data repository (Data Extraction and Analysis System (DEXA)). For analysis of % resistance, isolate-level data in the CIPARS AMR datasets were dichotomized into susceptible or resistant using current CIPARS breakpoints [28]. Minimum inhibitory concentration breakpoints can be found in Appendix B. As per routine CIPARS AMR analysis, to account for multiple isolates per farm, prevalence of individual and the two additional AMR outcomes (susceptible and resistance to ≥three antimicrobial classes (multiclass resistance)) were adjusted for clustering at the flock or herd level. Details of these analyses have been described elsewhere [28]. As an alternative to the routine methodology for estimating AMR prevalence, the flock- or herd-averaged AMR results were also determined. For each flock or herd, the total number of isolates classified as resistant to an antimicrobial of interest was divided by the total number of isolates for the flock/herd (one to four isolates depending on the recovery rates for poultry and one to six depending on the recovery rates for GF pigs) for that organism (*E. coli* or *Campylobacter*) for a specified year. The averaged AMR is interpreted as the proportion of flocks or herds with at least one isolate resistant (or susceptible) for the antimicrobial outcomes of interest. 

In *E. coli* and *Campylobacter*, respectively, percentages of resistance to the antimicrobials tested during the study timeframe using isolate-level and averaged flock or herd estimations are summarized in Appendix C. The prevalence levels and directionality of the shift in AMR (Appendix C Table A4 and Table A5) informed the selection of AMR outcomes for integration (Table 1).

For simplicity in describing the prevalence of resistance throughout the paper, the terms used in the EU/EEA for the reporting of AMR data were followed as a guide [32]: rare: <0.1%, very low: 0.1% to 1.0%, low: >1.0% to 10.0%, moderate: >10.0% to 20.0%, high: >20.0% to 50.0%, very high: >50.0% to 70.0% and extremely high: >70.0%. These do not pertain to MIC values and we caution our readers that these values are intended only as reference points and for descriptive purposes [32]. The authors recognize that certain antimicrobials, such as those that are categorized as highest priority critically important (HP-CIA’s) by the World Health Organization (WHO), even at rare or very low levels, could signify an emerging public health concern.

#### 2.2.3. Temporal Changes in AMR

The aim of temporal analysis was to provide insight on the directionality of the shifts and extent of the changes in resistance over specified time points. The relative change in the percentage resistance between two time points was determined (e.g., % GEN-R in 2019 minus % GEN-R in 2015). As per routine CIPARS temporal analysis [28], changes in AMR were determined with resistance (or susceptible) as binary outcome variable (Yes/No) with year as the independent categorical variable and *p* ≤ 0.05 was considered significant. An OR > 1 and OR < 1 indicates that probability of resistance increases or decreases, respectively, between the specified time points.

#### 2.2.4. AMR Outcomes for Integration and Rationale for Selection

##### 2.2.4.1. Homologous or Single Resistance Outcomes

Of the twelve and nine antimicrobials included in the NARMS Gram-negative and CAMPY plate configuration for *E. coli* and *Campylobacter*, respectively, six homologous antimicrobials were selected for inclusion in this analysis as summarized in Table 2.

##### 2.2.4.2. Additional Composite AMR Outcomes

Two composite AMR outcomes were also determined and used in AMU–AMR integration (Table 3): These complementary AMR outcomes are valuable for their simplicity (i.e., instead of reporting all homologous AMR outcomes) and provide a general indication on how the populations of bacteria, from resistant to susceptible, are shifting within the species or overall [31,32,46,47]. A strong inverse correlation between these two AMR outcomes was detected, which was consistent with the literature [31,32,46,47]. Alternate terminology for describing susceptible isolates includes “no resistance”, as seen in the US NARMS interactive data display [48], or its inverse value, resistance to at least one antimicrobial (i.e., used in some CIPARS communication products). Any of these AMR outcomes could be used to describe AMR trends for monitoring the progress of AMU stewardship initiatives. Susceptible and multiclass resistance outcomes were paired with total AMU to visually assess how susceptible isolates and multiclass resistance shifted in relation to total AMU over time.

In summary, the criteria for selection of AMR outcomes included public health importance, veterinary significance and the relevance of the measurement to total AMU and AMU stewardship. The rationale for selection for integration is synthesized in Table 1, Table 2 and Table 3. Temporal changes in AMR and the directionality of the shift between 2015 and 2019 are shown in Table 1. 

**Table 2:** Single or homologous antimicrobial resistance outcomes selected for integration.

AMR Outcome	About This AMR Outcome
Ceftriaxone resistance (CRO-R)	Organism: *E. coli* Indicator for resistance to: 3rd generation cephalosporins * Prevalence levels **: Low to moderate Rationale for inclusion: monitoring of Step 1 of the AMU reduction strategy in the poultry industry [49,50] ***. Closely monitored by other integrated surveillance system because of its public health significance [31,32,46,47].
Ciprofloxacin (CIP-R)	Organism: *Campylobacter* spp. Indicator for resistance to: fluoroquinolones * Prevalence: Moderate to high Rationale for inclusion: the detection of CIP-R *E.* *coli* was relatively very rare in the CIPARS farm AMR dataset. Therefore, CIP-R *Campylobacter* is used in routine monitoring and AMU–AMR data integration. Used for monitoring of the AMU reduction strategy in the poultry industry [49,50]. Closely monitored by other integrated surveillance system because of its public health significance [31,32,46,47]. Initial contamination of CIP-R could lead to a self-perpetuating cycle of CIP-R within farms [51].
Gentamicin resistance (GEN-R)	Organism: *E. coli* Indicator for resistance to: Aminoglycosides and aminocyclitols Prevalence: Low to high Rationale for inclusion: A compensatory increase in GEN-R because of aminoglycosides/aminocyclitol use in poultry was noticed following the voluntary elimination of the preventive use of ceftiofur in poultry in Canada. Flocks that used these antimicrobials were catgorized as high users of antimicrobials [24]. This antimicrobial class is included as part of a voluntary AMU reduction strategy in the poultry industry [49,50].
Trimethoprim and sulfamethoxazole (SXT-R)	Organism: *E. coli* Indicator for resistance to: trimethoprim and sulfonamides combination (inhibitors of folate synthesis) *. Prevalence: Low to moderate. Rationale for inclusion: flocks that used antimicrobials belonging to this antimicrobial class combination were categorized as high users of antimicrobials [24]. Resistance is monitored because the antimicrobial class is deemed as extra-label use in poultry [5,6].
Tetracycline resistance (TET-R)	Organism: *E. coli* Indicator for resistance to: tetracyclines Prevalence: High to very high Rationale for inclusion: commonly used in poultry and swine; flocks that used antimicrobials belonging to this class were categorized as high users of antimicrobials [24].
Azithromycin resistance (AZM-R)	Organism: *Campylobacter* spp. Indicator for resistance to: macrolides (plus lincosamides and streptogramin B). Prevalence: Low to very high Rationale for inclusion: correlates with erythromycin resistance. Macrolides are also part of the voluntary AMU reduction strategy in the Canadian poultry industry [49,50].

* The use of these antimicrobials is deemed as extra-label drug use (ELDU) in any poultry species in Canada. ** Please refer to Appendix C for prevalence estimates. *** Chicken Farmers of Canada. AMU strategy, a prescription for change [49] and Turkey Farmers of Canada. Guidelines for antimicrobial use in the turkey industry [50].

**Table 3:** Composite antimicrobial resistance outcomes selected for integration.

AMR Outcome	About This Composite AMR Outcome
Susceptible *E. coli* *	This includes isolates that exhibited susceptibility to all of the 14 antimicrobials included in the NARMS CMV4AGNF panel. For brevity, the term ”susceptible” is used to refer to these isolates. Rationale for inclusion: used for monitoring the progress of regulatory and voluntary changes in AMU in the food animal sector. As described in the literature, susceptible *E. coli* are an indicator used to assess the development of AMR in relation to total AMU in food-producing animals and are reflective of the overall AMR situation, including the status of *E. coli* carrying plasmid-mediated AMR genes [46].
Multiclass resistant *E. coli* (≥3 multiclass resistance)	Isolate resistant to individual antimicrobial classes was summed to provide the total number of classes that the isolate was resistant to. For brevity, the term “multiclass resistance” is used to refer to these isolates and represents isolates with resistance to ≥3 antimicrobial classes. Rationale for inclusion: complementary to the indicator above. As described in the literature, multiclass resistance is reflective of simultaneous actions of multiple antimicrobials on the indicator organism, therefore this outcome is also equally informative in detecting emerging AMR issues and is routinely used for AMR reporting in surveillance systems [31,32,46,47,52].

* It is important to note that isolates that exhibited susceptibility to antimicrobial concentration one dilution above the minimum inhibitory concentration breakpoint (i.e., deemed as isolates with reduced susceptibility/intermediate) were categorized as susceptible in the CIPARS AMR dataset.

### 2.3. AMU Data Component

Overview: As previously described, AMU data, including general farm-level demographics, biosecurity and animal health information, are captured in the questionnaires. These questionnaires were designed to generate high resolution data for reporting of complementary AMU metrics and indicators and to adapt to the evolving AMU reporting and communication methodologies.

#### 2.3.1. AMU Data Preparation

AMU indicators routinely used for the reporting of CIPARS farm-level AMU, as well as exploratory AMU indicators, were estimated at the flock- and herd-level prior to analyses and modelling exercises:

- Count-based: the percentage of use was determined (number of flocks or herds using an antimicrobial divided by the total flocks or herds multiplied by 100). The count-based AMU results were examined in relation to animal health data.
- Quantitative: the numerator and denominator input parameters are summarized in Appendix A and the formulae can be found in Appendix D. Throughout this manuscript, relevant descriptive statistics were used in the analyses and exploratory exercises. Description of the five candidate AMU indicators are summarized in Table 4.

#### 2.3.2. Temporal Analysis

**Figure A1:**
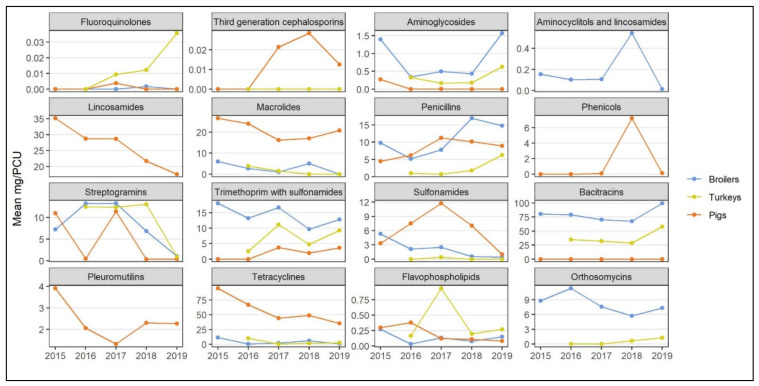
Temporal trends in the milligrams of antimicrobial by class per population correction unit by species for the period of 2015 to 2019 for broiler chickens and GF swine, and from 2016 to 2019 for turkeys.

- Count based: The first step involved visual inspection and the relative changes between two time points similar to AMR findings (i.e., percentage of flocks medicated with antimicrobial A in 2019 minus the % of flocks medicated with antimicrobial A in 2015) were determined. The second step involved temporal analysis using logistic regression models developed with AMU (Yes/No) as the binary outcome variable with year as a categorical independent variable and using *p* ≤ 0.05 for significance as previously described [28].
- Quantitative: trends in national-level AMU in the three species studied using the general AMU indicator used by CIPARS, mg/PCU, were visually inspected for levels of use and summarized in Appendix A Figure A1, organized by antimicrobial class. This provided an overall picture of the diversity of antimicrobial classes used and variations in temporal trends and quantity of use by antimicrobial class between the animal species in Canada. Relative changes in AMU quantity between two time points were descriptively assessed and summarized in Table 1 (directionality of the shift between 2015 and 2019).

Equation (1). Relative change in antimicrobial use quantity:

(1) $$\frac{AMU\;quantity\;in\;2019-AMU\;quantity\;in\;2015}{AMU\;quantity\;in\;2015}\times 100$$

**Figure 2:**
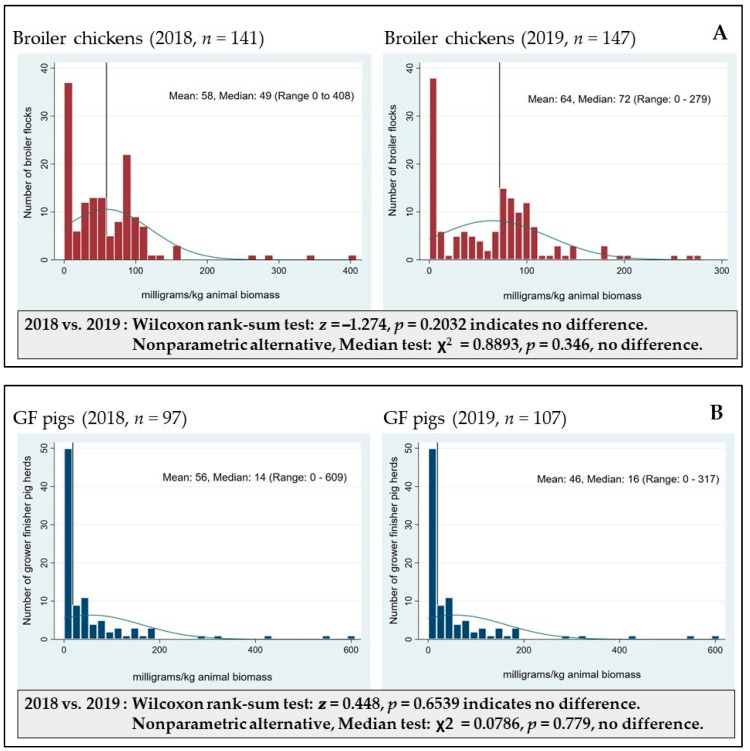

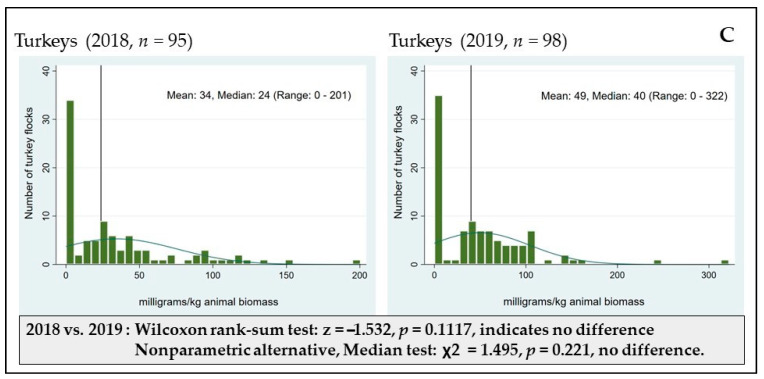
Case examples: visual inspection of the distribution of flock or herd level milligrams/kg animal biomass between two surveillance years (2018 and 2019). Broiler chickens (**A**). Grower-finisher (GF) pigs (**B**). Turkeys (**C**). Black lines pertain to the median mg/kg animal biomass. Two nonparametric tests were used to compare flock/herd distribution between two time points, and statistical measures are shown in the boxes underneath the figures.

In addition to the national data analysis (sum of AMU quantity adjusted for species biomass by year), the flock or herd level specific AMU values were determined and distributions of AMU were visually inspected. The asymmetrical shape of the AMU distribution curve was similar from year to year and across the three animal species studied. The analysis of the five candidate AMU indicators (not shown) yielded a similar shape which was also described in a previous study [24]. The skewed distribution signified various AMU practices reflective of changing production systems (i.e., zero AMU was organic, antibiotic free or raised without antibiotic flocks or herds). It is important to note that this asymmetrical distribution of flock or herd AMU is a relatively common observation in farm-level AMU surveillance systems across food animal species regardless of the AMU indicator used [22,54,55,56]. For visualization, only the mg/kg animal biomass, to harmonize with the OIE methodology, is presented in Figure 2A–C. Because of the shape of the AMU distribution, the nonparametric Wilcoxon rank sum test for comparing samples from relatively similar distributions was used. This approach evaluated the AMU distribution as a whole and ranked the number of flocks or herds according to their level of AMU (i.e., no use, moderate users and high users) then detected how these relative rankings shifted between time points. The procedure (RANKSUM in Stata SE/V16) returned the exact *p* values where ≤0.05 indicated statistical significance between two time points. In the example shown (between 2018 and 2019), the shape of the distribution curve of mg/kg remained similar between 2018 and 2019 and the *p* ≥ 0.05 indicated no significant difference between these years of surveillance. An alternate nonparametric test, the test of median (MEDIAN procedure in Stata SE/V16) noting the ꭓ^2^ and *p* values, was also explored, but because the results were comparable, the Wilcoxon rank sum test was deemed adequate for our circumstance and was used for further analysis. 

#### 2.3.3. AMU Outcomes Selected for Integration and Rationale

The total flock- or herd-level AMU and the six classes corresponding to the previously described AMR outcomes (3GC’s, FQs, AMGL-AMCL, MACR, TMPS and TET) in Table 2 were selected outcomes for integration. These antimicrobial classes are used for the prevention and treatment of the most frequently occurring diseases diagnosed in terrestrial food animals in Canada. As described in the AMR section, the elevated public health significance of the 3GCs and FQs also warrants closer monitoring of AMU in these classes. Total AMU has relevance for overall AMU stewardship (i.e., setting reduction targets). Considerations for inclusion in routine data integration are summarized in Table 1.

#### 2.3.4. AMU Indicator Selection

As part of integration, five candidate AMU indicators were evaluated for their utility for simultaneous evaluation of the trends in the AMU–AMR combined data, and to study the strength of the linkages between AMU and AMR by species. Three exercises were conducted.

##### 2.3.4.1. How the AMU Indicators Relate to Each Other

Two of the candidate AMU indicators were exploratory to CIPARS, the weight-based mg/kg animal biomass and the dose-based nDDDvetCA/kg animal biomass, which are derivatives of the mg/PCU and nDDDvetCA/PCU, respectively. To assess their relevance and ensure that these aligned with AMU indicators currently used by CIPARS, the pairwise correlation matrix was expanded from the previous constructed matrix [24] to include these two exploratory indicators. The correlation matrix was constructed for each species separately. This step was necessary to inform further selection of a candidate AMU indicator for the purposes of data integration, simultaneous assessment of AMU levels in the different species and the study of AMU–AMR associations. The correlation matrices analysis (Appendix D Table A6), regardless of the species, showed statistically significant high correlation between the two weight-based indicators, mg/PCU and mg/kg animal biomass (pairwise correlation coefficient, pcc = 0.86 to 0.99) and between the three dose-based indicators, nDDDvetCA/1000 animal-days at risk [TI_1000_], nDDDvetCA/PCU and nDDDvetCA/kg animal biomass (pcc = 0.83 to 0.99). These results indicated alignment of the exploratory AMU indicators with those routinely used by CIPARS for reporting and research.

##### 2.3.4.2. AMU Indicator for Comparison of Use between Species

**Figure 3:**
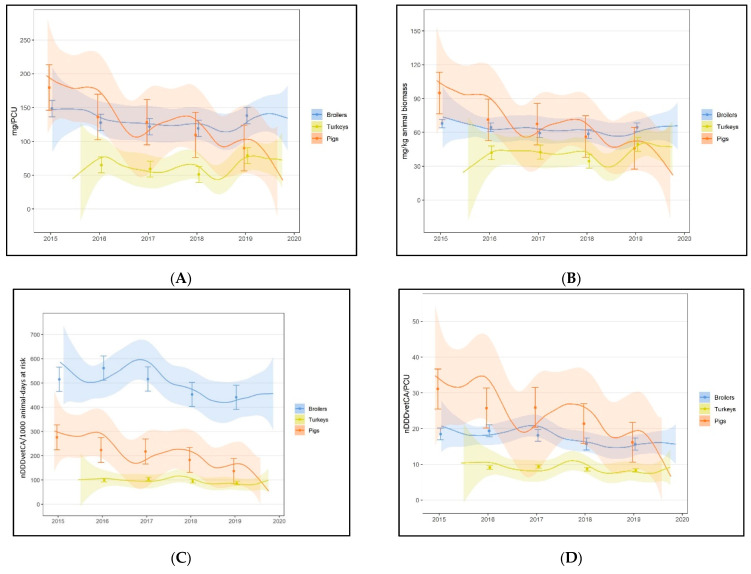

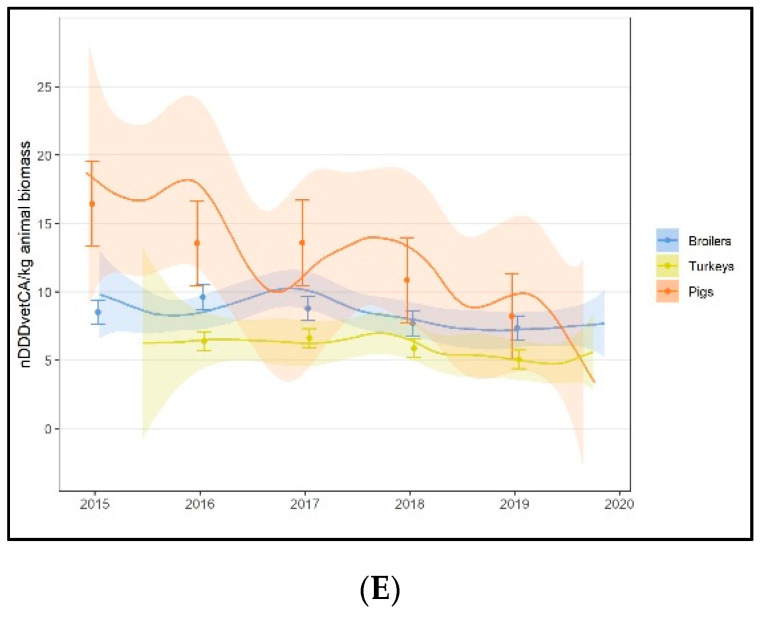
Spline curves for: visual inspection of the five candidate antimicrobial use (AMU) indicators to guide in the assessment of AMU indicators to be used for data integration. Milligrams per population correction unit (**A**). Milligrams per kg animal biomass (**B**). Number of defined daily doses using Canadian standards (nDDDvetCA) per 1000 animal-days at risk (**C**). nDDDvetCA/PCU (**D**). nDDDvetCA/kg animal biomass (**E**). National data collection for turkeys commenced in 2016. The plotted data points represent the annual flock or herd level means and the error bars indicate the standard deviation around these means. The surrounding ribbons represent the 95% CI around the curves. Readers are also encouraged to refer to Figure 2, which shows the flock and herd variations in AMU quantity.

Comparability of the AMU unit of measurement (metrics) between populations is a requirement if the intent of the study is to compare AMU levels between species [11]. Although a vast majority of the internationally recognized AMU indicators available to date are formulaically the same, certain metric/s (i.e., components of the formulae for an AMU indicator/input parameter) could impact the analytic outcomes and interpretation. To have a full picture of longitudinal trends and overall AMU levels in the species currently surveyed, and to guide in the assessment of the five candidate AMU indicators for their utility for integrated surveillance across species, spline curves patterned from the Netherlands annual AMU report in agricultural livestock [22] were constructed for each of the candidate AMU indicators. A smoothing spline was initially constructed with eight degrees of freedom; the number of degrees of freedom was chosen in order to allow approximately 1–2 knots per year of data, which optimized the visualization of longitudinal trends. Then, the spline was used to predict the AMU indicator over the five-year (broilers, GF pigs) or four-year (turkey) study timeframe. The number of degrees of freedom did not markedly sacrifice certainty around these predictions, which is represented as 95% CI banding around the spline curve; these data were plotted with flock or herd level mean and standard deviation around the mean (Figure 3). These constructed spline curves of flock or herd level data of the five AMU indicators showed that temporal trends varied depending on the species and the AMU indicator.

All of the AMU indicators examined established that GF pigs had the highest mean value, except in the nDDDvetCA/1000 animal-days at risk (Figure 3C), where broiler chickens exhibited higher values. For all AMU indicators, turkeys had the lowest mean values. The magnitude of the differences between species was highest in the nDDDvetCA/1000 animal-days at risk, as exhibited by the distinct separation of the broiler data from GF pig and turkey data. This could be explained by the differences in the input parameter, days at risk. This measurement is equivalent to the entire growing period (i.e., interpreted as the flocks or herds are at risk of being treated each day of the growing cycle that they are in the barns) [12]. The species with the shortest life span, broiler chickens (mean 35 days growing period), had the highest range of values for this indicator compared to GF pigs (mean 114 days) or turkeys (mean 89 days). As such, the days at risk in the input parameter for this AMU indicator resulted in substantial variations in the mean and range of nDDDvetCA/1000 animal-days at risk estimates between species. Attributes of the numerator input parameters (i.e., mg adjusted by the defined daily doses specific for each antimicrobial and animal species [53,57]) and denominator measurements (i.e., species-specific weights and number of animals per flock or herd) enabled better comparability of the annual mean and range of flock/herd AMU between species, thus nDDDvetCA/PCU and nDDDvetCA/kg animal biomass were the logical candidates for the simultaneous assessment of AMU levels and trends between species. Variations in the diversity of antimicrobials used depending on the species (which could impact total or class-specific nDDDvetCA’s) and the proportion of antimicrobial classes used have been described elsewhere [53]. The DDDvet/PCU has been utilized in several AMU research studies [17,24,58] and has been useful when other input parameters (i.e., the days at risk) for other more popular dose-based indicators, nDDDvetCA/1000 animal-days at risk, as above (TI_1000_), or the alternate indicator, TI_100_, are unavailable. Ultimately, for our study, the nDDDvetCA/kg animal biomass was selected for other study objectives (e.g., AMU–AMR association) and was preferred over nDDDvetCA/PCU to reduce the analytic burden of obtaining denominator data (i.e., average weight at treatment). Furthermore, slaughter live weights (alternate to pre-slaughter weight collected from farms as previously described) could also be conveniently obtained from processing plant records. In terms of communication to stakeholders, the kg animal biomass better aligns with other farm level production or economic parameters (total kg meat or live weight produced, total kg feed consumed). Additionally, this approach could be useful for estimation of dose-based AMU indicators in other food animal species where input parameters, such as weight at treatment, may not be easily accessed (beef, dairy, layer chickens) or may vary a great deal over the feeding period. In the Netherlands, a derivative of this indicator using annualized data (DDDA/Year) is used to compare the trends and levels of AMU across all the animal species in the country [22,59] and the AMU indicator used to study AMU–AMR associations [21]. 

##### 2.3.4.3. AMU Indicator for Studying the Relationship between AMU–AMR 

Multiple AMU indicators have been utilized for investigating the relationship between AMU and AMR, including the weight-based indicators, mg/kg animal biomass [39,60], mg/PCU [46,61], and the dose-based indicator, TI_100_ [62,63,64]. CIPARS currently utilizes some of these measurements for annual reporting of national AMU and descriptive assessments of AMU trends in relation to AMR.

**Figure 4:**
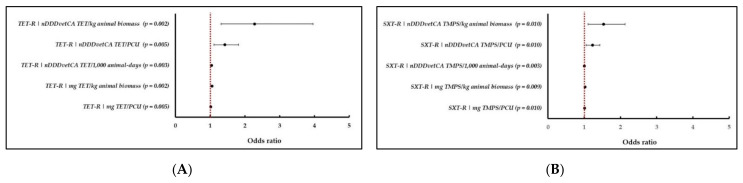
Case examples evaluating five antimicrobial use indicators for studying the potential associations between antimicrobial use and antimicrobial resistance using mixed effects logistic regression models. Tetracycline resistance (TET-R) and tetracyclines used in Turkeys (**A**). Trimethoprim and sulfamethoxazole resistance (SXT-R) and trimethoprim and sulfonamides use (TMPS) in grower finisher pigs (**B**) nDDDvetCA-number of defined daily doses in animals using Canadian standards. PCU-population correction unit. Red dotted vertical lines indicate an OR = 1 (no association between the antimicrobial use and resistance pairs). Horizontal lines indicate 95% Confidence Intervals around the OR (depicted by black solid dots).

For this exercise, two homologous AMU–AMR pairs were used as examples (Figure 4A,B). A homologous resistance (e.g., TET-R) was matched simultaneously with the five TET specific AMU indicators in turkeys. Resistance (Yes/No) to the specific antimicrobial of interest was the outcome variable, and one of the five AMU indicators of interest was the predictor variable. A new model was generated for each indicator. Forest plots were constructed to visualize the changes in the logistic regression outputs (OR’s, 95% CI’s and *p* values, referred to as effect estimates from this point forward). The model used is described in Section 2.4 (mixed effects logistic regression model with random slope for flocks/herds).

As demonstrated in the comparative analysis using the homologous pair, TET-R and TET use in turkeys (Figure 4A), all of the models resulted in significant associations and comparable *p* values (from 0.002 to 0.005), although the effect estimates substantially differed depending on the AMU indicator. For example, the nDDDvetCA TET/kg animal biomass and nDDDvetCA TET/PCU yielded higher OR with wider 95% CI’s, whereas the remaining indicators, nDDDvetCA TET/1000 animal-days at risk, mg TET/kg animal biomass and mg TET/PCU had comparable OR’s (1.02 to 1.05). Similarly (Figure 4B), using the data from GF pigs for SXT-R and TMPS use, the effect estimates varied depending on the AMU indicator. In this example, the dose-based indicators, nDDDvetCA TMPS/kg animal biomass and nDDDvetCA TMPS /PCU, yielded the highest OR’s with relatively wider 95% CI’s. These findings indicate that the interpretation of potential AMU–AMR relationships could change depending on the AMU indicator. However, we caution our readers that these observations are based only on the AMU indicators examined in this study. Furthermore, other AMU exposure parameters plausibly impacting AMR development including treatment duration (i.e., could vary by flock or herd and antimicrobial), route of administration and health of the animals at the time of treatment, were not determined in these exercises or not deemed as parameters in any AMU indicators used. These AMU attributes could be explored in more detail in future studies. Based on the forest plots and range of the OR estimates, the exercises confirmed that the nDDDvetCA/kg animal biomass also has utility for studying AMU and AMR associations. As described above, this indicator is methodologically similar with the indicator used by the Netherlands (annualized data) for monitoring levels of AMU and for investigating AMU and AMR across the different animal species [21,22]. Also, because dose-based indicators adjust for the average daily dose specific to the antimicrobial active ingredient and species, their utility for evaluating AMU–AMR has been suggested [11]. 

In total, eight AMU–AMR pairs (informed by the synthesis of results from routine surveillance as summarized in Table 1) were evaluated for potential AMU and AMR associations. The analysis of the association between the AMU–AMR pairs is beyond that which CIPARS routinely monitors (e.g., CRO-R and 3GC use). Assessment of multiple AMU–AMR pairs is indispensable, as this may signal potential compensatory use of an antimicrobial that could increase resistance to antimicrobials important to human health. For example, the elimination of the preventive use of 3GC’s led to the compensatory use of alternative antimicrobial classes including AMGL and aminoglycoside–lincosamide combination products. This shift in use resulted in increased isolation of GEN-R *E. coli* along the farm to retail continuum [65].

Box 1. **Decision: Which antimicrobial use indicator is appropriate for data integration within and across animal species?**

**Are the 5 AMU candidate AMU indicators related?** Yes, significantly moderate to high pairwise correlation coefficient (pcc) between weight based, and high pcc between dose-based AMU indicators.

(1) **For simultaneous evaluation of AMU levels (*and trends*) between species:**
  *Recommended:* unit of measurement/s comparable between species (11) *; dose-based (11) **.
  *Assessment:* Descriptive statistics by species (mean, 95% CI).
  *Candidate AMU indicators:*
  1) nDDDvetCA/kg animal biomass
  2) nDDDvetCA/PCU
(2) **For evaluating relationships between AMU and AMR**
  *Recommended:* unit of measurement reflective of the level and duration of exposure; dose-based.
  *Assessment:* mixed effect logistic regression model, evaluation of effect estimates (OR’s, 95% CI) by AMU indicator across species and forest plots visualization.
  *Candidate AMU indicators:*
  1) nDDDvetCA/kg animal biomass
  2) DDDvetCA/PCU
(3) nDDDvetCA/1000 animal-days at risk
  **Option selected for 1 and 2:** nDDDvetCA/kg animal biomass
  Other considerations for using this AMU indicator:
  (a) Reduced analytic burden in cases where input parameters such as the days at risk and average treatment weight (Option 3) are unavailable.
  (b) The denominator, average pre-slaughter weight, can be accessed from farm records or production summary reports. Alternate denominator such as processing weight and final birds/pigs slaughtered can be obtained from processing plant records.
  (c) Preferred for ease of communication with stakeholders (familiarity with the contextualizing denominator, e.g., kg live weight vs. PCU).

* adjusts for number of animals treated and weight ofanimals.
** adjusts for average daily dose per kg per day.

### 2.4. Development of Analytic Methods for AMU and AMR Integration

Overview: This section describes the integration of AMR and the quantitative AMU indicator selected from the exercises in Section 2.3 as described in Box 1 (nDDDvetCA/kg animal biomass). The flock or herd level AMU indicator was paired with the isolate-level AMR data, % resistance in *E. coli* and % resistance in *Campylobacter*, depending on the AMU–AMR pairs indicated in Table 1. 

In recent years, various research studies have explored the association between AMU and AMR. While the binary AMR measurements, % phenotypic resistance, were relatively common for the AMR component, various AMU data structures were used across different studies, for example, the use of binary AMU data [66,67,68,69] or continuous AMU data [21,39,60,61,62,63,64]. The high-resolution data collected by CIPARS enabled flexibility in AMU options, but for the purpose of this paper, the preferred AMU indicator nDDDvetCA/kg animal biomass (Box 1) was used for developing the methodology for evaluating AMR and AMU associations. Two logistic regression models were used to assess the strength of association between nDDDvetCA/kg animal biomass and the corresponding AMR outcomes.

- Option 1. Generalized linear model. The outcome of interest was the flock or herd level AMR for the antimicrobial and organism of interest. The strength of the association was evaluated using a generalized linear model (i.e., GLM procedure in Stata/SE V16.1; family–binomial distribution), with the flock- or herd-level AMR as the outcome variable (number of resistant isolates per flock and herd, accounting for the total isolates recovered per flock or herd), and the flock- or herd-level nDDDvetCA/kg animal biomass as the continuous predictor variable. The models were fitted for each animal species and adjusted for year (categorical variable).
- Option 2. Mixed effect logistic regression analysis. The second approach used the isolate-level AMR data matched with the corresponding flock or herd level AMU indicator, nDDDvetCA/kg animal biomass, as above. The models were fit for each animal species. For these data combinations, the strength of association was determined using mixed effect logistic regression (i.e., MELOGIT procedure in Stata/SE V16.1) adjusted for year, with a random intercept to account for the correlation of resistance within the same flock or herd.

Effect estimates (i.e., ORs, 95 CI’s and *p* values) were noted for each model and a *p* ≤ 0.05 was considered significant. An OR > 1 and an OR < 1 meant that AMR was positively or negatively associated with AMU, respectively. As an example, in the model using susceptible isolates as the outcome variable, an OR < 1 indicated that the probability of detecting isolates exhibiting susceptibility to all antimicrobials tested increased with lower AMU. For the broiler chicken data, all eight AMU–AMR pairs were modelled, but model convergence and unreliably wide 95% CI ranges were not achieved for some AMU–AMR pairs using the two modelling options above (i.e., due to rare outcomes or predictors). In succeeding species models, AMU–AMR pairs that had AMU values ≤0.1 nDDDvetCA/kg animal biomass or that had AMR levels categorized as rare or very low were not determined. 

**Figure 5:**
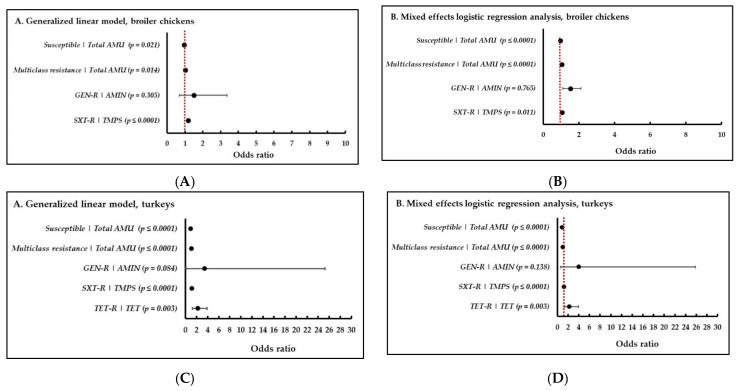
Case examples evaluating two analytic methodologies, generalized linear model (GLM) and mixed effects logistic regression model, for studying the potential associations between antimicrobial use and antimicrobial resistance. Broiler chickens using GLM (**A**). Broiler chickens, using mixed effects logistic regression model (**B**). Turkeys using GLM (**C**). Turkeys using mixed effect logistic regression models (**D**). GEN-R-gentamicin resistance, AMIN-Aminoglycosides, TET-R-tetracycline resistance, TET-tetracyclines, SXT-R-trimethoprim-sulfamethoxazole resistance, TMPS-trimethoprim and sulfonamides. Red dotted vertical lines indicate an OR = 1 (no association between the antimicrobial use and resistance pairs). Horizontal lines indicate 95% Confidence Intervals around the OR (depicted by black solid dots).

This exercise aimed to compare the utility of these two approaches to inform the methodology that could be routinely applied to farm-level surveillance data. To demonstrate how the two models explored in this study changed the effect estimates (i.e., OR’s, 95% CI’s and strength of association), forest plots were constructed (Figure 5). It is important to note that GLM and MELOGIT models yielded similar OR estimates but varied in the level of significance, as shown in Figure 5. In both approaches, antimicrobials with relatively low (<0.1) nDDDvetCA/kg animal biomass yielded unreliably wide 95% CI’s using either of the modelling procedures explored in this study, which was consistently observed in both broiler chickens and turkeys (e.g., GEN-R and AMGL). We caution our readers that some significant results may not be biologically important (e.g., persistence of resistance to certain antimicrobials in the absence of AMU selection pressure). On the other hand, nonsignificant results may have elevated public and animal health importance. As such, a review of complementary data (e.g., whole genome sequencing or detection of resistance determinants) and an in-depth assessment of other farm-level risk factors may also be conducted in parallel to these analyses. Additionally, exploration of other modelling approaches may be necessary for certain AMU–AMR pairs (i.e., very low quantity of use or rare AMR occurrence).

Both models have utility for further investigation of aspects of AMU exposures and farm-level factors that potentially have an impact on AMR, since the models could be expanded to include other risk factors for AMR. Mixed effects logistic regression was used for further analyses. In the future, inclusion of potentially relevant variables in the mixed effect logistic regression model, such as veterinary practices (i.e., potential random effects of veterinary practices) and geographical locations (i.e., similarities in production inputs such as hatcheries or poultry companies). As in other integrated surveillance systems [70], the methodologies explored in this present study could be refined over time to adapt to the rapidly evolving changes in AMU practices, where multiple modelling approaches or more than one AMU indicators may be necessary depending on the model fit/data available. In some cases, transformations of the data, for example, conversion of the mg antimicrobial to mg/1000 kg animal biomass [60] or log and quadratic transformations, may be necessary to obtain reliable effect estimates [70]. In this current study, the lognormal transformed nDDDvetCA/kg animal biomass indicated that OR estimates only changed slightly compared to the actual nDDDvetCA/kg animal biomass values, and model convergence was similarly not achieved in models with rare outcomes or predictors (e.g., CRO-R and 3GC pairs) or estimates in certain AMU–AMR pairs (e.g., GEN-R and AMGL). Other models, such as simple logistic regression models (LOGISTIC procedure in StataSE/V16), were also explored in this current study, and analyses yielded similar results (data not shown).

The strength of the CIPARS farm-level data is that both AMU and AMR data components were collected annually from the same epidemiological unit. Therefore, the isolates are more reflective of the actual farm-level AMU exposures, which is an advantage compared to other national surveillance programs. The data collection design (longitudinal in sentinel flocks and herds) is appropriate for measuring both the selection and spread of AMR [11]. Additionally, the high-resolution data collected from farms could be used to investigate other farm-level or industry-level operational factors that could impact AMR. Examples of studies using a subset of the farm-level data have been described elsewhere [39,71,72]. Future studies could also focus on an in-depth assessment of the impact of specific AMR mechanisms as they pertain to the different exposure characteristics, such as the spectrum of activity of the antimicrobials used, dosing requirements, the duration of exposure and the route of administration [11]. In summary, our proposed approach for species integration of AMU–AMR to be applied annually to the CIPARS farm data is indicated in Box 2.

Box 2. **Proposed methodology for species-level integration of AMU and AMR data.**

(1) Visual inspection, descriptive analysis of the percentages and directionality of the shift in AMU and AMR, followed by temporal analysis
  - **Simple logistic regression model:** AMR percentages, count-based AMU and animal health indicators
  - **Nonparametric, Wilcoxon rank sum test:** quantitative AMU indicators.
(2) Identify the AMU-AMR pairs:
  - public health importance
  - animal health significance
  - relevance to broader AMU stewardship and monitoring of AMU reduction efforts or other interventions
(3) Determine the strength of AMU-AMR association
  - mixed effect logistic regression approach
(4) Visualize the trends in AMU and AMR.
(5) Examine animal health data for context (*are trends in reported disease syndromes reflective of the trends in AMU*?).

### 2.5. Animal Health Context

Overview: The intent of this exercise was to incorporate the animal health data in order to provide context to the trends observed in AMU. This approach is meant to help improve our understanding of the impact of changing AMU practices due to species-specific voluntary [49,50] or federal regulatory changes, such as enhanced veterinary oversight [73,74]. It is also meant to identify any evolving preventive health practices with a so-called sparing effect on AMU, including vaccines/bacterins against specific agents and non-antimicrobial alternatives.

The CIPARS Farm questionnaire included a section on clinical impressions (i.e., syndromic surveillance) regarding species-specific disease syndromes and etiologic agents, where responses that were ‘likely positive’ or ‘confirmed positive’ were considered flock or herd positive for that disease syndrome or etiologic agent. Temporal changes in the percentages of flocks or herds that experienced any of these syndromes, and utilized preventive animal health programs (e.g., coccidiosis and necrotic enteritis control in poultry as shown in the complimentary paper, Part II-application) that may play a role in these syndromes, were determined to be similar to the AMU count-based indicator and AMR temporal analysis described in previous sections. The rationale for the selection of certain disease syndrome/etiologic agents was based on conditions that are commonly occurring in Canadian flocks and herds and that have economic significance due to the potential for high morbidity and mortality or reduction in production performance parameters, such as weight gain, feed conversion or cost of therapeutic AMU. The approach to disease syndrome/etiologic agents to be included has also been noted elsewhere, for example, a study in Europe that noted the impact of enteric diseases on flock performance [75]. For simplicity, in poultry, the disease syndromes/etiologic agents were broadly grouped into neonatal (caused by avian pathogenic *E.coli* (APEC)), enteric (*Clostridium perfringens* and *Eimeria* spp.), respiratory (APEC) and miscellaneous bacterial diseases (Table 5). In GF pigs, the most important economic disease syndromes/etiologic agents were *Streptococcus suis*, *Lawsonia* spp., *E. coli* and Swine Influenza Virus. It is acknowledged that laboratory confirmed data are more informative in understanding the impact of AMU as well as identifying disease syndromes/etiologic agents requiring preventive health interventions. The findings that the percentages of flocks or herds with reported clinical conditions significantly increased over time is concerning (Table 5). A more in-depth analysis of the impact of changing AMU practices on animal health and production should be further explored. Additionally, the characterization of susceptibility profiles in animal health pathogens, as highlighted in other surveillance systems, such as the United Kingdom’s Veterinary Antibiotic Resistance and Sales Surveillance Report [76] and Denmark’s DANMAP report [23], would contribute to the understanding of the impact of AMU changes on clinical pathogens. To substantiate the syndromic information collected from farm-level questionnaires, provincial and regional disease information [2,3] could be consulted to compare information on prevalent diseases affecting these species.

### 2.6. Summary, Species-Level Integration of Farm Surveillance Data

Section 2 discussed the species-level integration of AMU and AMR and the utility of the syndromic data for contextual information. The exercises described in this section will refine the synthesis of existing CIPARS Farm AMU and AMR data and provide better understanding of the current AMU and AMR situation in the Canadian food animal species included in CIPARS Farm Surveillance. The methodologies explored here will be applied in the future for routine assessment of AMU and AMR linkages, complementary to the current descriptive analysis and visualization of integrated data [57,77]. As in other surveillance systems [32,46,70], ongoing refinements would be necessary to continue to adapt to the rapidly changing methodology for data integration, analysis and reporting.

- AMU indicator/s to use: there is no single AMU indicator that can encompass every analysis objective, in particular for comparison of AMU between species (Section 2.3.4.2) and for studying AMU and AMR relationships (Section 2.3.4.3) However, the high-resolution CIPARS farm-level data enabled exploration of the utility of various AMU indicators for these various analysis objectives. In additi.on to being able to investigate various methodologies, the other advantage of the CIPARS farm surveillance design is that the collection of AMU and AMR information is from the same epidemiological unit (same flocks or herds). This is different than the situation in other countries, where data are collected at the national level and only ecological associations between AMU and AMR can be explored.
- Animal health context: the syndromic data collected from CIPARS farms also helps provide insights on the potential implications of changing AMU practices on the health of flocks/herds. More detailed analysis could be conducted in the future to assess the impact of the shifts in AMU on mortality and flock or herd production performance parameters, as well as the susceptibility profiles of the clinical pathogens.
- AMU–AMR associations: the analytic options presented here (e.g., GLM and mixed effects logistic regression models) were appropriate for use in routine annual analysis and reporting (adjustments to various levels of clustering such as within flocks/herds). It is important to note that there are many analytic methodologies described elsewhere, ranging from simple logistic regression analysis to more advanced methodologies where other relevant variables could also be included in the model (e.g., multilevel analysis accounting for other sources of variations in the data). Emerging methodologies could be continuously explored to refine the approach used here.

## 3. Methodology Development for Multispecies AMU–AMR Data Integration

**Figure 6:**
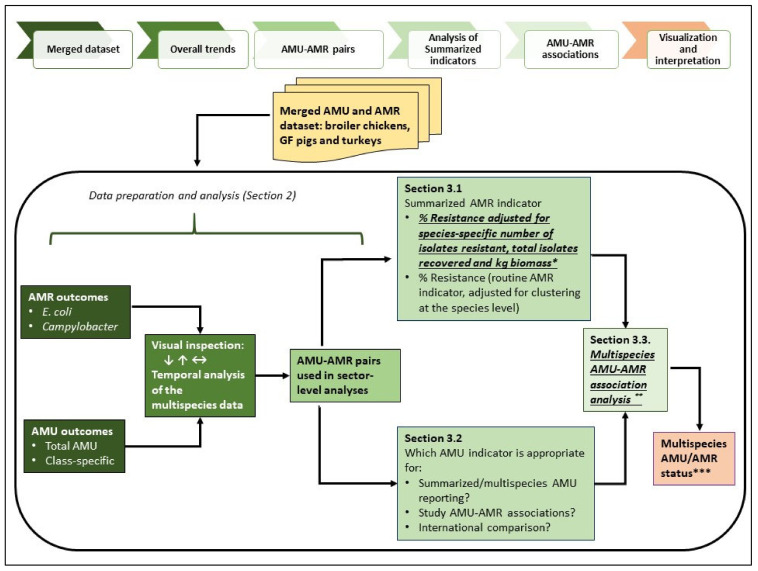
Approach multispecies-level integration of farm-level antimicrobial use and resistance data. AMU–antimicrobial use; AMR–antimicrobial resistance. Arrows indicate the directionality of the shifts in the national, five-year AMU, AMR and animal health data used in this study. Bold and underlined items represent exploratory analysis. * Candidate indicators included those that are routinely used by CIPARS and exploratory AMU indicators. ** Two-level random effects model (included random slopes for flocks or herds and species). *** Multispecies-level status of AMU and AMR.

For this paper, summarized reporting pertains to the use of aggregated AMU and AMR data from the three species included in this study. The intent of the exercises conducted in this section was to provide an overview of the current AMR and AMU situation in the animal sectors combined. Relevant analytic procedures determined from Section 2 were applied. It is envisaged that this methodology will be used to future CIPARS farm-level data analysis and will ultimately include other food animal species, currently in the early implementation or pilot phase (e.g., layer chickens, feedlot beef and dairy). The structured methodological steps for multispecies AMU-AMR integration is depicted in Figure 6.

### 3.1. AMR Summarized Indicator

Overview: The AMR levels across the three species by bacterial organism (*E. coli* and *Campylobacter* as summarized in Appendix C) were determined for each year using two AMR indicators as described below. The purpose of this exercise was to assess if the methods yield similar trends in resistance.

- % Resistance, multispecies: Overall % resistance in *E. coli* and *Campylobacter*, adjusted for clustering at the species level to account for similarities in AMR within animal species, was determined. Logistic regression analysis as described previously (i.e., isolate-level AMR as binary outcome variable and year as independent categorical predictor variable, and *p* ≤ 0.05 for significance) was used to detect temporal changes in overall % resistance.
- Animal biomass adjusted % resistance: The summarized AMR indicator developed in the EU/EEA, known as the AMR Indicator Index or Key Outcome Indicator [31,46,70], was determined. This AMR indicator was previously explored in another study using poultry data from CIPARS/FoodNet Canada [30], which was modified in this current study to also include AMR data from GF pigs and using kg animal biomass instead of PCU in the denominator. As shown in Equation (2), this AMR indicator accounts for the species-specific AMR results (total resistant isolates/total isolates per species) and fluctuations in animal biomass over time, such as when new herds or flocks are added to the sampling frame. Relative changes in the biomass adjusted AMR indicators were determined between two time points.

Equation (2). AMR weighted by species-specific percentage of resistant isolates and biomass

(2) $$\frac{R_{BrY\times }kg\;Broilers_{Y}}{kg\;animal\;biomass_{TotalY}}+\frac{R_{GFPigsY\times }kg\;GFPigs_{Y}}{kg\;animal\;biomass_{TotalY}}+\frac{R_{TkY\times }kg\;Turkeys_{Y}}{kg\;animal\;biomass_{TotalY}}=AMR_{BiomassAdj}$$

where: R_BrY_-% resistance (or susceptible) in broiler chickens (Appendix C); calculated per year from 2015 to 2019. R_GFPigsY_-% resistance (or susceptible) in grower-finisher pigs (Appendix C); calculated per year from 2015 to 2019. R_TkY_-% resistance in turkeys (Appendix C); calculated per year from 2016 to 2019. kg Broilers_Y_-kg broiler chicken biomass (number of broiler birds × average annual broiler pre-slaughter weight); calculated per year from 2015 to 2019. kg GF pigs_Y_-kg grower-finisher pigs’ biomass (number of GF pigs × average annual GF pigs pre-slaughter weight); calculated per year from 2015 to 2019. kg Turkeys_Y_-kg turkey biomass (number of turkeys × average annual turkey pre-slaughter weight); calculated per year from 2015 to 2019. kg animal biomass_TotalY_-combined animal biomass from the three species.

These exercises, exploring the utility of the two measures of AMR indicators and visual inspection, indicated that the directionality of the trends was similar regardless of the method used (as shown in Appendix D Table A7 and described in detail in a complementary paper, Part II–applications). With the relatively stable sampling frame, fluctuations in animal biomass may not impact the trends in AMR using the routine formula (unadjusted for animal biomass), however, with future expansion of the farm program to other species, the alternate analysis that includes adjusting for animal biomass could be further explored and may be more appropriate. Detailed assessment of the trends will be described in a complementary paper (Part II–application).

### 3.2. AMU Summarized Indicators

Overview: This section summarized the quantity of antimicrobials from the three animal species, adjusting for the animal biomass. One of the CIPARS objectives is to compare data with international surveillance systems that are using similar AMU measurements. For this purpose, mg/PCU [14] and mg/kg animal biomass [15] estimates during the five-year study timeframe were determined using Equations (3) and (4), respectively. As previously mentioned, the input parameters used in the denominators for Equations (3) and (4) were based on the actual data collected from the sentinel farms, as shown in Appendix A and formulae summarized in Appendix D, and differed from national or regional/global biomass estimation methodologies.

Equation (3). Summarized (total) mg antimicrobials weighted by overall PCU.

(3) mgBrY+mgGFpigsY +mgTkYPCUTotalY=mgPCU

where: mgBrY-total mg active ingredients in broilers; calculated per year from 2015 to 2019. mg_GFpigsY_-total milligrams active ingredients in GF pigs; calculated per year from 2015 to 2019. mg_TkY_-total milligrams active ingredients in turkeys; calculated per year from 2016 to 2019. PCU_TotalY_-the annual population correction unit in all flocks and herds surveyed, total population at risk multiplied by the ESVAC average weight at treatment (1 kg broiler chickens, 6.5 kg turkeys and 65 kg GF pigs); calculated per year from 2015 to 2019.

Equation (4) Summarized (total) mg antimicrobials weighted by overall kg animal biomass

(4) mgBrY+mgGFpigsY +mgTkYkg animal biomassTotalY=mgkg animal biomass

where: mgBrY-total mg active ingredients in broilers; calculated per year from 2015 to 2019. mg_GFpigsY_-total milligrams active ingredients in GF pigs; calculated per year from 2015 to 2019. mg_TkY_-total milligrams active ingredients in turkeys; calculated per year from 2016 to 2019. kg animal biomass_TotalY_–the annual kg animal biomass, the population at risk multiplied by the actual average live slaughter weights; calculated per year from 2015 to 2019.

The above measurements (Equations (3) and (4)) are logical choices for comparison with national sales and distribution data and other surveillance systems (e.g., ESVAC and OIE) that are using these indicators for annual reporting. It is important to note that these estimates pertain to the three species studied only, and other relevant food animal species, including beef, dairy, layers and minor species, were not yet included. 

### 3.3. AMU–AMR Association, Multispecies 

The multispecies analysis to study AMU–AMR linkages followed the approach used in Section 2. The overall AMU-AMU using the merged datasets (all species combined), and the same eight AMU–AMR pairs and indicators evaluated in the species-specific analyses were determined using mixed effects logistic regression, adjusted for year, and included a random intercept for species and flocks or herds. (i.e., MELOGIT procedure, two-level random effects model). The random effects parameters account for similarities in resistance patterns within the animal species and within flocks or herds (nested within species). Detailed results are described elsewhere (Part II–application). The multispecies integration is aimed to provide a synthesis of the data across the species surveyed, reflective of the AMU and AMR situation in the larger food animal sector. 

### 3.4. Summary, Multispecies-Level Integration of Farm Surveillance Data and Limitations

Section 3 discusses the multispecies-level integration of AMU and AMR reflective of the current AMU and AMR situation in the animal sectors sampled by CIPARS. The approach for multispecies integration will be used to assess the progress of the regulatory changes in AMU (e.g., enhanced veterinary oversight) implemented in the broader food animal sector in Canada. However, the data have certain limitations:

- AMU: in terms of comprehensiveness, the data included three food animal species, but other important commodities in Canada, such as beef and dairy cattle and chicken egg layers, were not included, as these data were not yet available at the time of the study.
- AMR: data were limited to Gram-negative indicator organisms for many of the analyses. Also, AMR of clinical pathogen data were not available.
- AMU–AMR analysis: other models (i.e., multivariate analyses) evaluating the full range of potential risk factors for AMR by species were also not completed as they were beyond the scope of this study; however, they could be explored in future research.
- Animal health: the addition of other production performance parameters that could substantiate stewardship efforts, such as feed conversion rates and final kg of meat produced (slaughter data), are not collected by CIPARS, but will be useful to include to provide a full understanding of the impact of AMU reduction efforts in the health, welfare and sustainability of the food animal sectors in Canada.

## 4. Conclusions

This paper explored two levels of AMU–AMR data evaluation, the species level and multispecies level, discussed the approach for analysis for each of the surveillance data components and highlighted the utility of syndromic data for providing context to the AMU trends; therefore, this study demonstrated how surveillance data could be optimized for informing surveillance best practices and AMU stewardship. It also emphasized that high resolution farm-level AMU data collection enables a surveillance program to adapt to rapidly evolving AMU surveillance methodology development, to study associations between AMU and AMR and to provide useful context for interpreting trends in AMU and AMR, thereby supporting antimicrobial stewardship activities and informing further research. Antimicrobial resistance surveillance in clinical pathogens, which is a component of other surveillance systems remains a gap; inclusion of this component in the CIPARS farm surveillance program will complement the animal health data collected. 

## Figures and Tables

**Table 1:** Summary of temporal trends in antimicrobial resistance and antimicrobial use outcomes in the animal species and considerations for integrated reporting.

	AMR Outcomes		AMU Outcomes		Considerations for Inclusion in Data Integration
	Increased	Stable or Decreased	Increased	Stable or Decreased	
Susceptible_Ecoli_|Total antimicrobials	Br *, GFP, Tk			Br, GFP *, Tk *	Primary indicators
≥3 MCR_Ecoli_|Total antimicrobials		Br *, GFP *, Tk		Br, GFP *, Tk *	Complementary indicators
CRO-R_Ecoli_|3rd gen. cephalosporins	Tk	Br *, GFP,		GFP, (Br)	WHO’s HP-CIA; HC-VDD Cat.1
GEN-R_Ecoli_|Aminoglycosides		Br, GFP, Tk	Tk *	Br *	WHO’s CIA’s; HC-VDD Cat.2
SXT-R_Ecoli_|Trimethoprim-sulfas	Br	GFP, Tk	Br, GFP	Tk	WHO’s HIA’s; HC-VDD Cat.2
TET-R_Ecoli_| Tetracyclines	Tk	Br *, GFP,	Tk	Br, GFP *	WHO’s HIA’s, HC-VDD Cat.3
CIP-R_Campy_|Fluoroquinolones	Br *, GFP *	Tk		(Br/GFP/Tk)	WHO’s HP-CIA; HC-VDD Cat.1
AZM-R_Campy_|Macrolides	Br, GFP	Tk		Br *, GFP, Tk *	WHO’s HP-CIA’s; HC-VDD Cat.2

* Species where statistically significant temporal changes between 2015 and 2019 (*p* ≤ 0.05) were detected; in parentheses–rarely used. Br–broilers; Tk–Turkeys; GFP–grower-finisher pigs; Susceptible-isolates that were susceptible or had intermediate susceptibility to the panel of antimicrobials used; ≥3 MCR-short for multiclass resistant isolates that exhibited a minimum inhibitory concentration above the breakpoints in antimicrobials belonging to at least 3 classes. R–resistant; CRO–ceftriaxone, GEN–gentamicin, SXT–trimethoprim sulfamethoxazole, TET–tetracycline, CIP–ciprofloxacin, AZM–azithromycin. AMR–antimicrobial resistance expressed in percentage of resistance; AMU–the antimicrobial use indicator, nDDDvetCA/kg animal biomass; WHO–World Health Organization; CIA–Critically-important antimicrobials; HIA–Highly important antimicrobials; HC-VDD–Health Canada’s Veterinary Drugs Directorate Categorization System (1 to 3).

**Table 4:** Description of the antimicrobial use indicators used.

AMU Indicator	About This AMU Indicator
Count-based	
Frequency	Percentage of flocks using a particular antimicrobial, or any antimicrobials for a specific disease syndrome or etiologic agent. Temporal trends were used to assess in parallel with animal health data.
Quantitative, weight-based	
mg/PCU	This is the general AMU indicator routinely used by CIPARS for reporting of AMU data (farm, national sales and distribution data). The PCU is based on the population size and average weight at treatment using either ESVAC weights [14] or Canadian weights at treatment.
mg/kg animal biomass	This indicator is used by the OIE for reporting global AMU data [15]; however, for this study, the kg animal biomass pertains to live weights documented immediately prior to the expected slaughter date and may differ from other biomass estimation methodology.
Quantitative, dose-based	
nDDDvetCA/1000 animal days at risk or Treatment Incidence 1000 (TI_1000_):	This is the dose-based indicator routinely used by CIPARS. The TI_I000_ is determined by species. This indicator expresses the number of doses a thousand animals would receive per day over the observation period. The observation period for CIPARS data is the grow-out period and denotes the days at risk. If instead of dividing by 1000, the nDDDvetCA is divided by 100 animal days at risk, the TI becomes TI_100_. This indicator is interpreted as the number of days an animal was treated with antimicrobials per 100 days, or the percentage of days treated [12,13]. The DDDvetCA values were developed for each of the species and the methodology and DDDvetCA standards are described elsewhere [29,53].
nDDDvetCA/PCU	This indicator is the total number of DDDvetCA’s adjusted for PCU. Interpreted as the mg adjusted for DDDvetCA standard for every PCU.
nDDDvetCA/kg animal biomass	This indicator is similar to nDDDvetCA/PCU where PCU is replaced with the kg animal biomass, and is interpreted as the mg adjusted for DDDvetCA standardfor every kg of live pre-slaughter weight. An alternate data source for the denominator is the final slaughter live weights obtained from processing plants.

AMU-antimicrobial use. PCU-population correction unit. ESVAC-European Surveillance for Veterinary Antimicrobial Consumption. nDDDvetCA-number of defined daily doses in animals using Canadian standards.

**Table 5:** Utility of animal health data for contextualizing trends in antimicrobial use: summary of temporal trends in animal health and antimicrobial use outcomes in the animal species using count-based measurements.

		Diagnosis		Antimicrobial Use	
Broad Syndromic Classifications	Examples of Specific Syndromes or Agents	Increased	Decreased	Increased	Decreased
Poultry					
Neonatal	Yolksacculitis, early septicemia	Br, Tk *			Br *, Tk *
Respiratory	Airsacculitis,	Br *, Tk *			Br, Tk
Enteric	Necrotic enteritis, coccidiosis, nonspecific enteritis	Br, Tk *			Br *, Tk *
Miscellaneous bacterial diseases	Vertebral canal osteomyelitis, *Staphylococcus* spp. Infection, clostridial dermatitis	Br *	Tk		Br
Swine					
Systemic	*E. coli* infections	GF pigs *			GF pigs
	*Lawsonia* spp.	GF pigs *			GF pigs *
	*Streptococcus suis*	GF pigs *			GF pigs
Respiratory	Swine Influenza	GF pigs *		GF pigs	

* Species where significant temporal changes (*p* ≤ 0.05) between 2015 and 2019 were detected. Br-Broilers, Tk-Turkeys, GF pigs-grower finisher pigs. Antimicrobial use pertains to the response to the question of whether they used any antimicrobial for that flock or herd.

## Data Availability

Data used in the exercises are included in the Appendix A, Appendix B, Appendix C and Appendix D and are presented in detail in Part II of this study (application).

## Abbreviations

AMU	Antimicrobial use
AMR	Antimicrobial resistance
CIPARS	Canadian Integrated Program for Antimicrobial Resistance Surveillance
DDDvetCA	defined daily doses using Canadian standards
ESVAC	European Surveillance for Veterinary Antimicrobial Consumption
OIE	World Organization for Animal Health
PCU	population correction unit
nDDDvetCA	number of defined daily doses using Canadian standards
TI	Treatment Incidence
UK VARSS	United Kingdom Veterinary Antibiotic Resistance and Sales Report
AMC	Amoxicillin-clavulanic acid
GEN	Gentamicin
AMP	Ampicillin
MEM	Meropenem
AZM	Azithromycin
NAL	Nalidixic acid
CHL	Chloramphenicol
SSS	Sulfisoxazole
CIP	Ciprofloxacin
STR	Streptomycin
CLI	Clindamycin
SXT	Trimethoprim-sulfamethoxazole
CRO	Ceftriaxone
TEL	Telithromycin
ERY	Erythromycin
TET	Tetracycline
FLR	Florfenicol
TIO	Ceftiofur
FOX	Cefoxitin
3GC’s	third generation cephalosporins
AMCL	aminocyclitol
AMGL	aminoglycosides
BAC	bacitracins
FQ	fluoroquinolones
LINC	lincosamides
MACR	macrolides
MLSB	macrolides, lincosamides and streptogramin B
PEN	penicillins
STRE	streptogramins
TET	tetracyclines
TMPS	trimethoprim and sulfonamides combination

## Appendix A

**Table A1:** Antimicrobial use numerator and denominator input parameters and number of isolates from broiler chickens, grower-finisher pigs and turkeys used for this study, 2015 to 2020.

	2015	2016	2017	2018	2019	Mean	SD
Broiler chickens							
Number of flocks	135	136	137	141	147	139	4
Numerator parameters (AMU quantity)							
Total kg	448	396	407	469	495	443	34
Total nDDDvetCA’s ('000)	56,254	59,195	58,248	63,290	55,707	58,539	2461
Denominator parameters							
Population, n birds	3,035,442	3,052,498	3,212,784	3,794,167	3,474,669	3,313,912	262,233
PCU (*n* birds × 1 kg)	3,035,442	3,052,498	3,212,784	3,794,167	3,474,669	3,313,912	262,233
Average pre-slaughter live weight, kg	2	2	2	2	2	2	0
Total broiler chicken biomass, kg	6,302,129	6,006,219	6,464,738	7,563,080	7,239,121	6,715,057	536,875
Average days at risk	35	34	34	34	35	35	0
Number of samples	544	544	548	560	588	557	17
Number of isolates							
*Escherichia coli*	539	543	539	547	571	548	12
*Campylobacter*	117	93	122	122	142	119	16
Grower-finisher pigs							
Number of herds	85	91	82	97	107	92	8
Numerator parameters (AMU quantity)							
Total kg	1704	1110	984	1235	1197	1246	223
Total nDDDvetCA’s ('000)	292,705	214,175	169,554	183,335	196,013	211,156	39,567
Denominator parameters							
Population, n pigs	148,696	147,795	130,829	149,693	169,894	149,381	11,314
PCU (*n* pigs × 65 kg)	9,665,240	9,606,675	8,503,885	9,730,045	11,043,110	9,709,791	735,411
Average pre-slaughter live weight, kg	124	125	126	127	126	126	1
Total GF-pigs biomass, kg	18,126,786	18,235,934	16,293,023	18,609,944	21,314,478	18,516,033	1,472,631
Average days at risk	113	114	114	114	115	114	0
Number of samples	510	552	492	594	641	558	55
Number of isolates							
*Escherichia coli*	500	544	484	585	628	548	53
*Campylobacter*			369	483	447	433	48
Turkeys							
Number of flocks		72	74	95	98	85	11
Numerator parameters (AMU quantity)							
Total kg		219	210	211	374	253	62
Total nDDDvetCA’s ('000)		31,705	32,973	36,719	37,883	34,820	2285
Denominator parameters							
Population, n birds		558,396	550,587	608,994	687,360	601,334	48,740
PCU (*n* birds × 6.5 kg)		3,629,571	3,578,812	3,958,461	4,467,840	3,908,671	316,813
Average pre-slaughter live weight, kg		10	10	10	10	10	0
Total turkey biomass, kg		5,011,422	5,070,026	5,582,249	6,353,551	5,504,312	481,420
Average days at risk		90	89	87	89	89	1
Number of samples		280	292	371	399	336	51
Number of isolates							
*Escherichia coli*		277	287	367	393	331	50
*Campylobacter*		171	157	191	214	183	21
Combined total							
Number of sentinel flocks and herds	220	299	293	333	352	299	41
Numerator parameters (AMU quantity)							
Total kg	2151	1725	1600	1916	2066	1891	188
Total nDDDvetCA’s ('000)	348,959	305,075	260,776	283,343	289,603	297,551	26,823
Denominator parameters							
Population, n animals	3,184,138	3,758,689	3,894,200	4,552,854	4,331,923	3,944,360	435,009
PCU, total	85	31,796	33,055	36,816	37,990	27,948	12,889
Total animal biomass, kg	1,704	559,505	551,570	610,229	688,557	482,313	245,210
Number of samples	1054	1376	1332	1525	1628	1383	195
Number of isolates							
*Escherichia coli*	1039	1364	1310	1499	1592	1361	189
*Campylobacter*	117	264	648	796	803	526	283

The input parameters above were surveillance level totals for each data type (AMU or AMR); some analysis used in this study required the exclusion of a few observations and may differ from values previously reported by CIPARS data (i.e., flocks or herds missing either data from questionnaires or AMR results).

## Appendix B. Minimum Inhibitory Concentration

**Table A2:** Resistance breakpoints in *E. coli*.

Panel Type: CMV4AGNF			
Antimicrobial	Susceptible	Intermediate	Resistant
Amoxicillin/Clavulanic Acid ^1^	≤8	16	≥32
Ampicillin ^1^	≤8	16	≥32
Azithromycin ^2^	≤16	-	≥32
Cefoxitin ^1^	≤8	16	≥32
Ceftriaxone ^1^	≤1	2	≥4
Chloramphenicol ^1^	≤8	16	≥32
Ciprofloxacin ^1^	≤0.06	0.12–0.5	≥1
Gentamicin ^1^	≤4	8	≥16
Meropenem ^1^	≤1	2	≥4
Nalidixic Acid ^1^	≤16	-	≥32
Sulfisoxazole ^1^	≤256	-	≥512
Tetracycline ^1^	≤4	8	≥16
Trimethoprim/Sulphamethoxazole ^1^	≤2	-	≥4

^1^ CLSI guideline M100, 31th ed. Wayne, PA: Clinical and Laboratory Standards Institute; 2021. ^2^ No CLSI breakpoints. Breakpoints based on distribution of MIC’s and were harmonized with NARMS.

**Table A3:** Resistance breakpoints in Campylobacter.

Panel Type: Cmvcampy			
Antimicrobial	Susceptible	Intermediate	Resistant
Gentamicin ^2^	≤2	4	≥8
Clindamycin ^2^	≤2	4	≥8
Azithromycin ^2^	≤2	4	≥8
Erythromycin ^1^	≤8	16	≥32
Florfenicol ^2^	≤4	-	-
Ciprofloxacin ^1^	≤1	2	≥4
Meropenem ^2^	≤1	2	≥4
Nalidixic Acid ^2^	≤16	32	≥64
Tetracycline ^1^	≤4	8	≥16

^1^ CLSI guideline M45, 3rd ed. Wayne, PA: Clinical and Laboratory Standards Institute; 2016. ^2^ No CLSI breakpoints. CIPARS Clinical breakpoints. CLSI: Clinical and Laboratory Standards Institute. NARMS: National Antimicrobial Resistance Monitoring System. CIPARS: Canadian Integrated Program for Antimicrobial Resistance Surveillance.

## Appendix C. Antimicrobial Resistance Summaries

**Table A4:** Temporal variations in resistance of *Escherichia coli* isolates from three food animal species sampled at the farm level, 2015 to 2019.

Year	Isolate Level Analysis (Adjusted for Clustering)					Flock or Herd-Level Analysis (Mean Prevalence)				
	2015	2016	2017	2018	2019	2015	2016	2017	2018	2019
Broilers										
Number of isolates/flocks	539	543	539	547	571	134	136	137	140	147
Ampicillin	42%	40%	38%	32%	32%	47%	48%	50%	46%	40%
Ceftriaxone	12%	9%	10%	7%	7%	12%	9%	10%	7%	7%
Gentamicin	19%	21%	20%	20%	17%	19%	21%	20%	21%	17%
Nalidixic acid	6%	5%	5%	10%	8%	6%	5%	6%	10%	8%
Streptomycin	46%	48%	50%	46%	40%	47%	48%	50%	46%	40%
Tetracycline	54%	48%	48%	42%	39%	54%	48%	47%	42%	39%
Trimethoprim-sulfamethoxazole	16%	16%	17%	12%	15%	16%	16%	18%	12%	14%
Susceptible isolates	22%	24%	28%	32%	34%	22%	24%	28%	33%	34%
Multiclass resistance	40%	37%	39%	33%	31%	40%	37%	39%	34%	31%
GF pigs										
Number of isolates/herds	500	544	484	585	628	85	92	80	99	107
Ampicillin	30%	33%	28%	29%	29%	30%	33%	28%	28%	28%
Ceftriaxone	2%	2%	0%	2%	2%	2%	2%	0%	2%	2%
Gentamicin	1%	1%	1%	2%	1%	1%	1%	1%	2%	1%
Nalidixic acid	0%	0%	0%	1%	0%	0%	0%	0%	1%	0%
Streptomycin	45%	42%	42%	43%	38%	45%	42%	42%	43%	39%
Tetracycline	67%	70%	68%	67%	65%	67%	70%	68%	67%	64%
Trimethoprim-sulfamethoxazole	12%	13%	15%	12%	12%	13%	13%	15%	12%	12%
Susceptible isolates	23%	21%	23%	22%	22%	22%	21%	24%	22%	22%
Multiclass resistance	38%	39%	38%	36%	31%	39%	39%	37%	36%	30%
Turkeys										
Number of isolates/herds		277	287	367	393		70	73	93	100
Ampicillin		30%	37%	29%	29%		30%	38%	29%	29%
Ceftriaxone		1%	1%	1%	2%		1%	1%	1%	2%
Gentamicin		20%	24%	14%	11%		20%	24%	14%	11%
Nalidixic acid		1%	2%	1%	2%		1%	2%	1%	2%
Streptomycin		48%	51%	38%	39%		48%	51%	38%	39%
Tetracycline		69%	63%	56%	61%		69%	62%	56%	62%
Trimethoprim-sulfamethoxazole		9%	9%	11%	10%		9%	9%	10%	10%
Susceptible isolates		25%	25%	31%	28%		25%	26%	31%	28%
Multiclass resistance		36%	39%	26%	28%		36%	39%	26%	28%

**Table A5:** Temporal variations in resistance of Campylobacter isolates from three food animal species sampled at the farm level, 2015 to 2019.

	Isolate Level Analysis (Adjusted for Clustering)					Flock or Herd-Level Analysis (Mean Prevalence)				
	2015	2016	2017	2018	2019	2015	2016	2017	2018	2019
Broilers										
Number of isolates	117	93	122	122	142					
Azithromycin	18%	0%	5%	0%	1%	18%	0%	6%	0%	1%
Ciprofloxacin	16%	13%	18%	12%	24%	16%	13%	18%	12%	24%
Tetracycline	56%	22%	40%	27%	27%	56%	22%	40%	27%	27%
GF pigs										
Number of isolates			369	483	447					
Azithromycin			44%	40%	41%			43%	40%	40%
Ciprofloxacin			8%	11%	12%			9%	12%	13%
Tetracycline			67%	64%	63%			67%	64%	63%
Turkeys										
Number of isolates		171	157	191	214					
Azithromycin		1%	16%	8%	5%		1%	16%	8%	6%
Ciprofloxacin		22%	30%	38%	37%		21%	31%	38%	36%
Tetracycline		43%	49%	41%	43%		43%	46%	41%	43%

Values in red fonts on the right-hand side of the table (grouped AMR data by flock or herd) are prevalence values that deviated by ≥1% (maximum = 3%) from the isolate-level analysis on the left-hand side of the table.

## Appendix D. Methods Used to Calculate Antimicrobial Use for Surveillance Data Collected from Farms

**Measurements**	**Description and Reference**	**Numerator**	**Denominator**
**Count-Based Indicator, National-Level Values**			
1. Frequency	Yes, No data from the questionnaire.	No. of flocks or herds using antimicrobials	Total no. of flocks or herds surveyed
		***Other count-based measures: disease syndromes reported, vaccines, preventive health measures***	
**Weight-based indicators, flock or herd level**			
2. mg/PCU	Generic AMU measurement developed by ESVAC [14], used by CIPARS [24,28] in reporting the farm and national sales/distribution data and research studies	Total flock or herd: mg of all classes By class: mg antimicrobial class	Broilers: Birds at risk × 1 kg ESVAC average weight at treatment GF pigs: Pigs at risk × 65 kg ESVAC average weight at treatment Turkeys: Birds at risk × 6.5 kg ESVAC average weight at treatment
3. mg/kg animal biomass	CIPARS exploratory analysis: used by OIE for reporting the global OIE AMU database [15]. Actual pre-slaughter weights collected on farm at close to market were used in the study.	Total flock or herd: mg of all classes, as in #2 By class: mg antimicrobial class	Broilers: Birds at risk × kg broiler biomass GF pigs: Pigs at risk × kg GF pigs biomass Turkeys: Turkeys at risk × kg turkey biomass
**Dose-based indicators, flock or herd level**			
4. nDDDvetCA/1000 animal days at risk	Also known as treatment incidence (TI_1000_,) when values are divided by 100, it is called TI_100_; routine dose-based AMU indicator for reporting the CIPARS farm data and research studies	Total flock: nDDDvetCA’s of all classes By class: nDDDvetCAs, antimicrobial class	Broilers: Birds at risk × 1 kg ESVAC average weight at treatment × days at risk GF pigs: Pigs at risk × 65 kg ESVAC average weight at treatment × days at risk Turkeys: Birds at risk × 1 kg ESVAC average weight at treatment × days at risk
		***Values were multiplied by 1000 or 100 for TI_100_*** [12,13]	
5. nDDDvetCA/PCU	Used in past CIPARS research and reports [24,30,34] and in research studies and found to be highly correlated to the indicator in 4 above.	As in indicator 4 above	As in indicator 2 above.
6. nDDDvetCA/kg animal biomass	Explored previously [24]: used actual kg live pre-slaughter weight in the denominator instead of PCU	As in indicator 4 above	As in indicator 3 above.
nDDDvetCA-number of defined daily doses for animals using Canadian standards; this is the mg active ingredient divided by the DDDvetCA standard developed for each species and antimicrobial active ingredient [53]. ESVAC-European Surveillance for Veterinary Antimicrobial Consumption. PCU-population correction unit.			

**Table A6:** Correlation matrices by species using two weight-based and three dose-based indicators.

	mg/PCU	mg/kg Animal Biomass	TI1000	nDDDvetCA/PCU	nDDDvetCA/kg Animal Biomass
Broiler chickens					
mg/PCU	1				
mg/kg animal biomass	0.9115 *	1			
TI_1000_	0.7959 *	0.7933 *	1		
nDDDvetCA/PCU	0.8320 *	0.7529 *	0.9758 *	1	
nDDDvetCA/kg animal biomass	0.7380 *	0.8126 *	0.9741 *	0.9211 *	1
GF pigs					
mg/PCU	1				
mg/kg animal biomass	0.9976 *	1			
TI_1000_	0.8943 *	0.8921 *	1		
nDDDvetCA/PCU	0.8976 *	0.8943 *	0.9904 *	1	
nDDDvetCA/kg animal biomass	0.8970 *	0.8982 *	0.9889 *	0.9976 *	1
Turkeys					
mg/PCU	1				
mg/kg animal biomass	0.8592 *	1			
TI_1000_	0.7981 *	0.7507 *	1		
nDDDvetCA/PCU	0.8310 *	0.6859 *	0.9729 *	1	
nDDDvetCA/kg animal biomass	0.6589 *	0.7815 *	0.9263 *	0.8318 *	1

* Significant pairwise correlation coefficient *p* ≤ 0.005. The nDDDvetCA/1000 animal days at risk or TI_1000_, when adjusted by 100 (TI_100_), showed the same coefficient and *p* values.

**Table A7:** Summarized AMR indicator (combined species data), comparison between routine analysis (% resistance) and summarized reporting (% resistance adjusted for animal biomass).

AMR Outcome	Year	% Resistance	% Resistance, Biomass Adjusted	Difference Between AMR Adjusted and Unadjusted Values
Susceptible	2015	22%	23%	1%
	2016	23%	22%	−1%
	2017	25%	25%	−1%
	2018	28%	26%	−2%
	2019	28%	26%	−2%
Multiclass resistance	2015	39%	36%	−3%
	2016	38%	38%	1%
	2017	39%	38%	0%
	2018	33%	34%	1%
	2019	30%	32%	1%
Ceftriaxone	2015	7%	5%	−3%
	2016	5%	3%	−1%
	2017	4%	3%	−2%
	2018	3%	3%	0%
	2019	4%	3%	−1%
Gentamicin	2015	10%	6%	−4%
	2016	13%	9%	−4%
	2017	14%	10%	−4%
	2018	11%	8%	−3%
	2019	9%	6%	−3%
Trimethoprim–sulfamethoxazole	2015	14%	13%	−1%
	2016	13%	13%	0%
	2017	15%	14%	0%
	2018	12%	12%	0%
	2019	13%	12%	0%
Tetracycline	2015	60%	63%	3%
	2016	61%	65%	4%
	2017	59%	62%	4%
	2018	55%	59%	4%
	2019	55%	59%	4%
Ciprofloxacin	2017	14%	14%	1%
	2018	11%	16%	5%
	2019	13%	19%	6%
Azithromycin	2017	22%	30%	8%
	2018	24%	25%	1%
	2019	34%	26%	−7%
